# Evaluation of Potential Reference Genes for Relative Quantification by RT-qPCR in Different Porcine Tissues Derived from Feeding Studies

**DOI:** 10.3390/ijms12031727

**Published:** 2011-03-07

**Authors:** Qimeng Li, Konrad Johann Domig, Thomas Ettle, Wilhelm Windisch, Christiane Mair, Karl Schedle

**Affiliations:** 1 Institute of Animal Nutrition, Products, and Nutrition Physiology (APN), University of Natural Resources and Life Sciences, 1190 Vienna, Austria; E-Mails: thomas.ettle@lfl.bayern.de (T.E); christiane.mair@boku.ac.at (C.M.); qimeng.li@boku.ac.at (Q.L.); 2 Department of Food Sciences and Technology, Institute for Food Science, University of Natural Resources and Life Sciences, 1190 Vienna, Austria; E-Mail: konrad.domig@boku.ac.at (K.J.D.); 3 Chair of Animal Nutrition, Technische Universitaet Muenchen-Weihenstephan, Liesel-Beckmann-Strasse 6, 85350 Freising-Weihenstephan, Germany; E-Mail: wilhelm.windisch@wzw.tum.de (W.W.)

**Keywords:** sus scrofa, RT-qPCR, reference genes, gene stability

## Abstract

Five potential reference genes for RT-qPCR application, namely histone H3, beta-actin, GAPDH, ubiquitin and 18S rRNA, were evaluated for normalization of gene expression in four selected tissues (liver, kidney, thyroid and abdominal fat). Tissues were derived from fattening pigs exposed to different amounts and type of dietary iodine. Two software applications (*geNorm* and *NormFinder*) were used to evaluate the stability of the potential reference genes. All studied genes displayed high expression stability but different stability patterns between the investigated tissues. The results suggest GAPDH and 18S rRNA as reference genes applicable in all tissues investigated. Beta-actin and histone H3 are suitable reference genes for all tissues investigated except fat. In contrast, ubiquitin should be excluded from use as a reference gene in the porcine tissues analyzed due to variations in expression levels, despite the good expression stability.

## 1. Introduction

Iodine is an essential trace element for humans and animals. Iodine deficiency disorders like goiter, cretinism, reproductive failure or hypothyroidism are highly abundant. This is due to native iodine contents in feeds and food, often being too low to match requirements. Hence, efforts have been taken to fortify feedstuffs with iodine. Currently, the maximum permitted iodine content to add to pig feed has been set to 10,000 μg I/kg within the EC. Modern feeding practice actually fully utilizes this legal range to a high degree [1]. In context with high dietary iodine supply to pigs, the rise in iodine content of pork meat has often been highlighted as a beneficial side effect for human nutrition [2]. However, data from pigs as well as from laboratory rodents indicate a concomitant accumulation of iodine in tissues [3], the biological impact on the organism remains unknown.

The reverse transcription real-time PCR (RT-qPCR) technique provides the possibility to detect effects of iodine administration on the organism at the mRNA level. For relative quantification of the mRNA using the RT-qPCR technique, the activity of a target gene is expressed in relation to a reference gene (RG) in order to exclude general variations in transcript levels of the cell. Appropriate candidates for RGs should show stable and unregulated expression in the tissue sample under investigation [4]. Hence, before a gene may be chosen as reference, an exhaustive search is needed to ensure that no significant regulation occurs. However this can be a circular problem, as the expression data of the tested standard, as well, has to be standardized [5].

Numerous papers reported that beta-actin, glyceraldehyde-3-phosphate dehydrogenase (GAPDH), histone H3, ubiquitin, and 18S rRNA are expressed constitutively and are involved in basic reference functions required for cell maintenance [6–10]. Thus, they are commonly used as endogenous internal controls to normalize gene expression in molecular biology studies. However, precondition for their proper use are constant expression levels among different cells of one tissue and different types of tissues, as well as stability of expression levels against varying ambient factors such as experimental treatments (e.g., nutritional challenges). Nevertheless, it is important to note that constitutive expression in cells does not preclude regulation [11]. Furthermore, transcript levels of RGs may vary between different types of tissue and under different ambient conditions acting on the entire organism, as has been demonstrated, e.g., for tissues derived from experimental animal studies [12–20].

In 2009, Bustin *et al.* [21] published the minimum information for publication of RT-qPCR (MIQE guidelines). Many former RT-qPCR studies dealing with RGs did not provide the required information in the material and methods section. In this context, the aim of the present study was to investigate the stability of five potential RGs (histone H3, beta-actin, GAPDH, ubiquitin and 18S rRNA) in different tissues derived from nutritionally challenged fattening pigs, using two software applications (*geNorm* and *NormFinder*) to analyze a single data set under consideration of the MIQE guidelines.

## 2. Results and Discussion

### 2.1. RNA Quality and Integrity

RNA quality and integrity was controlled to prove the suitability of the sample material for RT-qPCR: The determination of OD_260/230_ and OD_260/280_ ratios produced mean values of 1.99 ± 0.07 and 1.67 ± 0.52, respectively (liver 2.03 ± 0.02 and 1.91 ± 0.17; kidney 2.04 ± 0.05 and 1.96 ± 0.23; thyroid 1.97 ± 0.04 and 1.71 ± 0.41; fat 1.90 ± 0.07 and 1.10 ± 0.59). Samples of different tissues showed a mean 28S/18S ratio of 0.87 ± 0.25 (liver 0.97 ± 0.13, kidney 0.82 ± 0.20, thyroid 0.75 ± 0.37, fat 0.93 ± 0.12).

### 2.2. Gene Expression Results

No template control and no RT-control were negative, indicating suitable PCR runs.

When comparing the results of both software applications, *geNorm* and *NormFinder* produced identical ranking orders of stability (Table 1 and Table 2). All genes studied reached a high expression stability with M values (average expression stability) below the default limit of *M* = 1.5 [22]. However, different ranking orders of reference genes between the investigated tissues were observed (Table 1 and Table 2). Ubiquitin showed comparably low *M* and stability values in all tissues. Thus, it always ranked first or second position. In contrast, histone H3 showed the highest M and stability values in abdominal fat tissue and liver. In liver and abdominal fat, GAPDH and beta-actin, respectively, were ranked in the first position.

The results of the present study are in accordance with previous studies, where variation in gene expression stability between different types of tissue from the same organism has been reported [22–24]. In porcine liver, our study as well as other authors [25,26], showed that the commonly used RG GAPDH was the most stable one, whereas in kidney GAPDH proved to be more unstable. Hence, evaluation of tissue specific RGs is essential.

Ubiquitin showed significantly different relative expression rates (*p* < 0.05) between all investigated tissues (data not shown). For histone H3 and beta-actin an up-regulation in fat tissue was observed between the treatment groups (*p* < 0.05). Additionally, treatment specific expression in liver was detected for ubiquitin (*p* < 0.05). Variations in expression levels of potential RGs in relation to type of tissue and ambient conditions (e.g., experimental treatments) [27,28] gave rise to the necessity to use a set of more than one RG [22].

## 3. Experimental Section

### 3.1. Animals and Tissues

The tissue samples were derived from a feeding trial with fattening pigs (*n* = 15 × 4), exposed to different amounts (400 *vs.* 4000 mg iodine per kg of feed) and chemical composition (iodide *vs.* iodate) of added dietary iodine in a 2 × 2 factorial arrangement. Animals were housed under common practical conditions and were fed conventional diets modified only in view of the iodine supply levels and sources. At the end of fattening (mean body weight approximately 112 kg), the animals were slaughtered under standardized conditions.

Samples from liver (left lobe of the liver), kidney (marrow), thyroid and abdominal fat tissue were isolated from the pig carcass. The tissue samples were immediately snap frozen in liquid nitrogen and stored at −80 °C until total RNA extraction starting three month after slaughtering.

### 3.2. One Step RT-qPCR Methods

Approximately 50 mg of tissue sample was homogenized in 0.5 mL TriFast^™^ reagent (PeqLab, Germany) with a Precellys 24 homogenizer (PeqLab, Germany). Total RNA from liver tissue samples was isolated using TriFast (Peqlab, Germany) applying the method of Fleige *et al.* [29]. In comparison to the general RNA extraction method [29], the homogenization time was increased by 10 s and only 30 μL DEPC-dH_2_O (Sigma Chemical Co, USA) was applied to dissolve the RNA pellet in kidney tissue. Thyroid tissue samples were homogenized at 6,500 rpm for 15 s, thereafter further 0.5 mL of TriFast^™^ reagent was immediately added to the homogenate and then incubated for 5 min at room temperature. 200 μL chloroform was added into each tube, 400 μL of upper aqueous solution was transferred to a sterile microtube (VWR, Germany) with equivalent volume of isopropanol. Finally, only 30 μL DEPC-dH_2_O was added to the RNA pellet. All other steps were performed according to the general RNA extraction method. Fat tissue samples were homogenized at 5000 rpm for 10 s, centrifuged (Centrifuge 5810R, Eppendorf, Germany) thereafter at 12,000 rpm for 15 min at 4 °C and incubated for 5 min at room temperature. 200 μL chloroform was added into each tube, 300 μL of upper aqueous solution was transferred to a sterile microtube with equivalent volume of isopropanol. Finally, 20 μL DEPC-dH_2_O was added to the RNA pellet. The other steps were kept the same as in the general RNA extraction method.

To quantify the extracted RNA concentration, the optical density was determined using a UV spectrophotometer (NanoDrop Technologies, USA). OD_260/230_ and OD_260/280_ ratios were checked considering sample purity. In addition, RNA integrity was analyzed by a micro-fluidic capillary electrophoresis in the Experion system (Bio-Rad Laboratories, USA). From each tissue investigated, extracted total RNA of high quality of six samples per treatment was used for RT-qPCR.

Until RT-qPCR total RNA was stored for three weeks at −80 °C. RT-qPCR analysis was performed using the primers shown in Table 3. Primer for GAPDH, 18S rRNA and ubiquitin were designed with Primer 3 software (http://frodo.wi.mit.edu/), histone H3 and beta-actin were obtained from literature [30,31]. Amplification was carried out as one-step PCR with the Corbett Rotor-Gene^™^ 3000 (Corbett, Australia) using the QuantiFast^™^ SYBR^®^ Green RT-qPCR Kit (Qiagen, Germany). The PCR reaction consisted of 5 μL 2 × QuantiFast SYBR Green PCR Master Mix, 0.5 μL each for forward and reverse primers (10 pmol), 3.8 μL diluted template RNA (10 ng/μL), 0.1 μL QuantiFast RT Mix and 0.1 μL RNase-free water in a total volume of 10 μL. The following cycling protocol was used: 10 min at 50 °C, 5 min at 95 °C, followed by 40 cycles of 10 seconds at 95 °C and 30 s at 60 °C. To verify consistency of the amplicon, the product was tested in a melting point analysis conducted directly after amplification, for determining dissociation of the PCR products from 65 °C to 95 °C. Data on mRNA expression for each sample and gene analyzed were obtained as quantification cycle (*C*_q_) and single run efficiencies (*E*), using the Rotor-Gene 3000 software version 6.0 (Corbett, Australia). To clarify that samples were free of genomic DNA, a negative RT-control was performed. Additionally, a no template control was included in the PCR runs.

### 3.3. Data Evaluation

Of each treatment group six samples with the highest 28S/18S ratio were used for statistical analysis and a single-run-specific, efficiency-corrected expression model was applied [32]. Data were transferred into Excel-based files and *geNorm* [22] and *NormFinder* [33] were used to identify the most appropriate RG. The GLM procedure of SAS (SAS Inst., Inc., Cary, NC) was used to determine treatment and tissue effects by analysis of variance. A *p*-value of <0.05 was regarded as significant.

## 4. Conclusions

We report on an experiment that was performed in order to identify appropriate reference genes to be used for relative gene quantification by RT-qPCR in pigs using MIQE standards. Not one single RG always manifests stable expression levels in all tissue types under investigation, thus emphasizing the necessity to characterize the suitability of various RGs to serve as internal controls in the respective tissue type where transcription effects are tested. Our study suggests 18S rRNA and GAPDH as RGs applicable on all tissues investigated. Beta-actin and histone H3 are suitable RGs for all tissues investigated except fat, whereas ubiquitin should be excluded from use as a RG in the porcine tissues analyzed due to variations in expression levels, despite the good expression stability. The present results should act as an addition to similar studies such as Nygard *et al.* [24] and can be used as a guideline for future feeding studies with trace elements applying the pig as an animal model.

## Figures and Tables

**Table 1.** Candidate reference genes according to the expression stability (calculated as the average M value after stepwise exclusion of worst scoring genes) by the *geNorm* VBA applet.

	**Liver**		**Kidney**		**Thyroid**		**Abdominal Fat**	

Ranking Order	Gene Name	Average *M* value	Gene Name	Average *M* value	Gene Name	Average M value	Gene Name	Average *M* value
1	GAPDH	0.846	Ubiquitin	0.760	18S rRNA	0.948	Beta Actin	0.764
2	Ubiquitin	0.876	Histone H3	0.820	Ubiquitin	0.975	Ubiquitin	0.894
3	18S rRNA	1.040	Beta Actin	0.835	Histone H3	0988	18S rRNA	0.950
4	Beta-actin	1.071	GAPDH	0.860	Beta Actin	1.072	GAPDH	0.990
5	Histone H3	1.115	18S rRNA	0.971	GAPDH	1.536	Histone H3	1.051

**Table 2.** Candidate reference genes for normalization of RT-qPCR listed according to their expression stability calculated by *NormFinder* VBA.

	**Liver**		**Kidney**		**Thyroid**		**Abdominal Fat**	

Ranking Order	Gene Name	Stability Value *	Gene Name	Stability Value *	Gene Name	Stability Value *	Gene Name	Stability Value*
1	GAPDH	0.258 (±0.082)	Ubiquitin	0.291 (±0.071)	18S rRNA	0.281 (±0.096)	Beta Actin	0.133 (±0.101)
2	Ubiquitin	0.318 (±0.081)	Histone H3	0.391 (±0.077)	Ubiquitin	0.368 (±0.095)	Ubiquitin	0.407 (±0.081)
3	18S rRNA	0.569 (±0.099)	Beta Actin	0.396 (±0.078)	Histone H3	0.381 (±0.096)	18S rRNA	0.491 (±0.088)
4	Beta-actin	0.584 (±0.100)	GAPDH	0.425 (±0.080)	Beta Actin	0.534 (±0.106)	GAPDH	0.538 (±0.093)
5	Histone H3	0.627 (±0.105)	18S rRNA	0.557 (±0.094)	GAPDH	0.989 (±0.159)	Histone H3	0.601 (±0.100)

* The numbers in brackets denote the standard error for the stability value.

**Table 3.** Details of primers used for each of the five evaluated genes.

**Gene**	**Accession No.**		**Sequences[5′→3′]**	***T*_M_**	**Product length [bp]**
GAPDH *sus*	AF017079	for	gccatcactgccacccagaa	60 °C	153
		rev	gccagtgagcttcccgttga		
Ubiquitin *sus*	U72496	for	accctgacgggcaagaccat	60 °C	143
		rev	cggccatcctccagctgttt		
Histone H3 *sus*	NM_213930	for	actggctacaaaagccgctc	60 °C	232
		rev	acttgcctcctgcaaagcac		
Beta-actin *bov*	AY141970	for	aa***c***tccatcatgaagtgtgacg *	60 °C	229
		rev	gatccacatctgctggaagg		
18S rRNA *sus*	DQ437859	for	tggagcgatttgtctggtta	60 °C	200
		rev	acgctgagccagtcagtgta		

* incorrect sequence in forward primer (real CDS sequence in AY141970 aa***t***tccatcatgaagtgtgacg).
